# Some Spinal Cord Lesions.—III

**Published:** 1911-07-22

**Authors:** 


### NEUROLOGY.

### SOME SPINAL CORD LESIONS.—III.

### INSULAR, MULTIPLE, OR DISSEMINATE SCLEROSIS.

This is a chronic disease of the brain and spinal cord, and the chief symptoms which characterise it are a peculiar tremor and inco-ordination of the hands, arms, and head, nystagmus, and scanning speech. Localised areas of connective tissue are found in the brain or spinal cord or in both, which partly or entirely replace the normal nerve elements in these situations.

The onset is slow and insidious. Weakness and stiffness of the legs may be the earliest manifestations of the disease; in fact, unless a careful and systematic physical examination is made, the condition may be mistaken for primary spastic paraplegia, even when it is well advanced, and before this it is apt to be mistaken for hysteria in a woman or for unsteadiness from drink in a man.

In a well-marked case the following may be noted: there is generally weakness and rigidity of the legs, together with tremors in the arms, legs, and head on making any purposive movement. As a rule there is no weakness of the arms, nor is there any tremor so long as they are at rest; but on attempting any particular movement, such as picking up an object from a table, the hands and arms at once begin to tremble and oscillate in an irregular manner. The tremor is therefore described as volitional or intentional. When lying in the recumbent position no tremor is noticeable, but on sitting up the head begins to shake. The tremor becomes very evident in the handwriting, for the outline of each letter is shaky and irregular, and in advanced cases the writing becomes so irregular as to be illegible. In some cases writing is altogether impossible.

Another good method of demonstrating the tremor and inco-ordination is to give the patient a glass of water to drink; on attempting to raise the glass to the lips it is so much shaken that a considerable amount of water may be spilled.

As a rule there is no anæsthesia, but there is frequently impairment of the muscular sense, which may be demonstrated by directing the patient to close his eyes and then to touch the end of his nose with the tip of either index finger.

The knee-jerks are increased and ankle clonus and extensor plantar reflexes may be elicited. The radial and ulnar wrist-jerks and the tricipital and bicipital elbow-jerks may be unduly brisk; the abdominal reflex may be lost early. The grey matter of the cord is less frequently affected than the white matter, so that muscular atrophy is an unusual symptom.

The electrical reactions are usually normal. The sphincters are not involved until the disease is well advanced, except in quite unusual cases. The speech is occasionally scanning or staccato in character, *i.e.* words are pronounced slowly and the syllables are accentuated. Nystagmus, either lateral or vertical, is one of the most constant and characteristic signs of the disease. Oculo-motor paralyses are rare. Amblyopia from optic atrophy is not uncommon. The absence of headache, vomiting, and optic neuritis serves to exclude cerebellar tumour, and in the presence of increased knee-jerks and extensor plantar reflexes, the occurrence of well-marked nystagmus points clearly to disseminated sclerosis, even if there be neither intention tremor nor staccato speech.

Combined Degenerations of the Spinal Cord.

This is a sub-acute disease characterised by the simultaneous occurrence of degeneration in tracts of different function in the spinal cord. It is frequently associated with severe anæmia, and in some cases it has followed influenza and other acute diseases, including syphilis. Women between the ages of thirty and fifty are more commonly affected than men. The lesions are most marked in the mid-dorsal region of the cord, and consist of degenerative changes in the white matter with the exception of a narrow area in immediate contact with the grey matter which is also unaffected.

The onset is gradual, the earliest symptoms being tingling and numbness in the toes and fingers. Shooting pains may be experienced in the legs and a girdle pain around the waist. The gait is spastic, and there is unsteadiness when the patient stands with the eyes shut and the feet close together.

In a well-marked case the following points would be noticed: the legs are weak and rigid, and as the disease progresses they become flaccid and much weaker.

There may be paræsthesia of the feet and legs. In advanced cases there is anæsthesia and analgesia. The knee-jerks are exaggerated, there are extensor plantar reflexes, and ankle clonus. As the disease advances the knee-jerks become abolished, but the plantar reflex remains extensor in character, and this association is one of the most important diagnostic features of the disease. The muscles of the legs ultimately become much wasted. There is a diminished reaction to the interrupted current; on testing with the continuous current, although the contraction is diminished, there is no polar change.

